# Predictive validity of the National Benchmark Test and National Senior Certificate for the academic success of first-year medical students at one South African university

**DOI:** 10.1186/s12909-020-02059-8

**Published:** 2020-05-13

**Authors:** Sfiso Emmanuel Mabizela, Ann Zeta George

**Affiliations:** Centre for Health Science Education, Faculty of Health Sciences, University of the Witwatersrand, Phillip V Tobias Health Sciences Building, 29 Princess of Wales Terrace, Parktown, Johannesburg, 2193 South Africa

**Keywords:** First-year academic success, Medical students, Selection tests, Hierarchical multiple regression, National Benchmark Test, National Senior Certificate, South Africa

## Abstract

**Background:**

South African medical schools use the results of the National Senior Certificate (NSC) examination for selecting students. Five of the nine medical schools also use the National Benchmark Test (NBT). The University of the Witwatersrand weights the NSC and NBT results equally in the selection process. This study addresses the predictive validity of the NBT and NSC for academic success. The association between the NBT proficiency levels and students’ progression outcomes was also investigated.

**Methods:**

Data obtained from the University’s Business Intelligence Services for 1652 first-year medical students from 2011 to 2017 were analysed using hierarchical regression models and chi-square tests. The three NBT domains and four of the NSC subjects were the independent variables in the regression models, with the first-year grade point average for students who passed the first year as the dependant variable. The NBT performance levels and first-year progression outcome (passed, failed, or cancelled) were used in the chi-square analysis. Frequency tables were used to describe the cohort’s demographic details and NBT results. Crosstabs were used to analyse student performance according to the school quintile system.

**Results:**

The three NBT domains explained 26% of the variance, which was statistically significant, *R*^2^ = 0.263, *F* (3, 1232) = 146.78, *p* < 0.000. When the NSC subjects (Life Sciences, English, Mathematics, and Physical Science) were added to the regression equation, they accounted for an additional 19% of the variance, *R*^2^ = 0.188, *F* (3, 1229) = 137.14, *p* < 0.000. All independent variables contributed 45% of the variance, *R*^2^ = 0.451, *F* (6, 1229) = 166.29, *p* < 0.000. A strong association between the NBT proficiency levels and first-year students’ progression outcomes was observed.

**Conclusion:**

The NBT results, when weighted equally to the NSC results, explained more variance than the NSC alone in predicting academic success in the first year of the medical degree. The NBT should not only be used for selecting medical students but should also be used to place students with lower entry-level skills in appropriate foundation programmes and to identify students who are admitted to regular programmes who may need additional support.

## Background

The first-year performance of medical students is considered to have a profound influence on their future academic progress [1]. A successful first year promotes the development of a positive attitude, self-confidence, and a commitment to their studies [2]. Admission tests are used to identify students with cognitive abilities to cope with the intellectual demands of the medical programme and non-cognitive attributes to assimilate the ethical, inter-relational, and motivational challenges [3, 4]. All nine South African medical schools use the results for selected subjects from the National Senior Certificate (NSC) in their selection process. The NSC is written in the final year (Grade 12 or the matric year) of the Further Education and Training level.^1^ Five medical schools use both the NSC results and the National Benchmark Test (NBT) to select students [3].

The NSC results represent the extent to which a student has met the requirements for grades R–12 [5], based on the final examinations. For each subject, students are allocated rating codes from 1 (not achieved) to 7 (outstanding achievement), which correspond to a range of scores expressed as percentages (see Table 1). Based on their NSC results, students obtain either a Higher Certificate, National Diploma, or a Bachelor Degree pass (see Table 2), which determine the higher education training programmes students are eligible to pursue.

**Table 1:** NSC rating codes and descriptions [5]

Rating code	Description	Score
7	Outstanding achievement	80–100
6	Meritorious achievement	70–79
5	Substantial achievement	60–69
4	Adequate achievement	50–59
3	Moderate achievement	40–49
2	Elementary achievement	30–39
1	Not achieved	0–29

**Table 2:** NSC outcome descriptors [6]

Higher Certificate	Pass the NSC with at least a rating of 2 (30–39%) for the language of learning and teaching of higher education institutions.
National Diploma	Pass the NSC with an achievement rating of 3 (40–49%) or better in four subjects. At least a rating of 2 (30–39%) for the language of learning and teaching of the higher education institution.
Bachelor Degree	Pass the NSC with an achievement rating of 4 (50–59%) or better in four subjects from the designated list. At least a rating of 2 (30–39%) for the language of learning and teaching of the higher education institution.

Higher Education South Africa^2^ commissioned the NBT in 2005, to complement the NSC results and to provide universities with information about first-year university students’ entry-level skills [7, 8]. The NBTs are written in the final year (grade 12) of the Further Education Training level by prospective university entrants, depending on the admission requirements of the degree they intend studying. Universities use the NBT results to select and place students in programmes for which they qualify. The NBT assesses students in three domains: NBT Mathematics (NBT MAT), NBT Academic Literacy (NBT AL) and NBT Quantitative Literacy (NBT QL). The NBT results provide information about students’ abilities and skills that could predict their future academic performance [9]. The NBT AL tests academic reading and reasoning abilities, aiming to examine students’ abilities to engage successfully with the language demands of higher education [10, 11]. The NBT QL examines students’ abilities to solve quantitative problems (mathematical and statistical) in areas relevant to higher education programmes [11]. The NBT MAT test is designed to assess students’ understanding of mathematical concepts from the school Mathematics curriculum and disciplines such as Mathematics, physics and chemistry [11, 12]. The NBT MAT is written by those students who intend applying for programmes that require Mathematics [13]. Students’ performance in each domain of the NBT is categorised into four levels (*proficient, intermediate upper, intermediate lower, and basic*), for universities to make selection choices (Table 3).

**Table 3:** NBT benchmarks set in 2015 for degree purposes [14]

NBT level	Domain [percentage range]	Recommended programmes
Proficient	NBT MAT [69–100] NBT AL [68–100] NBT QL [70–100]	Performance suggests that academic performance will not be adversely affected in cognate domains. If admitted, students should be placed in regular programmes of study.
Intermediate upper	NBT MAT [52–68] NBT AL [54–67] NBT QL [55–69]	Students are likely to need complementary support (additional tutorials, workshops, augmented courses, language intensive work).
Intermediate lower	NBT MAT [35–51] NBT AL [40–53] NBT QL [40–54]	Students need to be placed in an extended programme.
Basic	NBT MAT [0–34] NBT AL [0–39] NBT QL [0–39]	Test performance reveals serious learning challenges: it is predicted that students will not cope with degree-level study without extensive and long-term support. Institutions admitting students performing at this level would need to provide such support themselves.

The NSC is norm-referenced while the NBT is criterion-referenced [15]. Norm-referenced tests differ from criterion-referenced tests in their purpose, content selection, and nature of scoring, which influences how the results are interpreted [16]. The NSC is designed to sort and rank students, and the NBT is intended to show students’ performance in clearly defined domains that require mastery [16, 17].

The South African higher education system is afflicted by low throughput rates and increasing failure and dropout rates [11, 18, 19]. The poor university throughput rate could be partially explained by the inequities that persist in the Basic Education system. Several authors, e.g. Prince [15], Walton, Bowman and Osman [20], and Maringa and Osman [21], have described South African Basic Education as effectively consisting of two schooling systems. The two disparate systems are believed to contribute to racialised representation and throughput at university [20–22].

South African schools are classified using a quintile system based on socioeconomic indicators, including the average income and unemployment rates in a school’s geographical location [23]. Quintile one is the poorest quintile, quintile two is the second-poorest quintile, and so on [24]. Each quintile encompasses one-fifth of the learners enrolled in public ordinary^3^ schools [24]. Teachers with the most substantial content knowledge tend to teach in quintile 5 schools [22]. Initially intended to provide funding for schools in lower socio-economic areas, the quintile system introduced by the Department of Education in 2006 is regarded as having reinforced socioeconomic inequalities [23]. The less affluent schools in quintiles 1, 2, and 3, which include many of the former Black^4^ and Coloured^4^ schools, tend to produce learners with limited abilities in reading, writing, and numeracy [20, 25], while the schools in quintiles 4 and 5, largely made up of the former White^4^ and Indian^4^ schools, produce more university entrants and graduates [20–22].

The Faculty of Health Sciences at the University of the Witwatersrand (Wits University) ranks applicants according to a composite index (CI) that weights the NBT and NSC results equally [9]. The four other South African medical schools that use the NBT for selection purposes use NBT weightings of 30 and 40% [3]. The 50% NSC contribution to the CI used at Wits University is derived from applicants’ marks in English, Mathematics, Physical Science or Life Sciences, and the subject with the highest mark of the remaining subjects, except for the subject, Life Orientation. All three of the NBT domains are used to calculate the CI.

In response to government imperatives to produce more healthcare professionals and to reflect the country’s demographics, South African medical schools have adjusted their admission requirements [3]. Currently, 40% of the places available in the Bachelor of Medicine and Bachelor of Surgery (MBBCh) degree at Wits University are reserved for top-performing students. The remaining 60% is divided equally into three categories: top-performing rural students, top-performing students from quintile 1 and 2 schools, and top-performing Black^4^ and Coloured^4^ students. Wits University introduced rurality as a selection criterion in 2015.

The teaching programme for the six-year MBBCh degree at Wits University is divided into clinical and pre-clinical years [4]. The medical curriculum is shown in Table 4. The first 2 years focus on the basic sciences [4]. The third and fourth years are the beginning of the clinical years, structured within integrated system-organ blocks [9]. The fifth and sixth years are the clinical years during which students are allocated to clinical clerkships in four academic hospitals [9].

**Table 4:** Medical curriculum at Wits University

Year of study	Subject
1	Introduction to Medical Sciences I Chemistry I Physics I Sociological Foundations of Health Psychological Foundations of Health System Dynamics for Medical Students
2	Human Anatomy Molecular Medicine Physiology and Medical Biochemistry I Medical Thought and Practice I
3	Integrated Basic Medical and Human Sciences A
4	Integrated Basic Medical and Human Sciences B
5	Integrated Clinical Medicine A
6	Integrated Clinical Medicine B

Tutoring programmes are available to all students across all years of study who need help with individual subjects. In addition, the Faculty’s Office of Student Success (OSS) identifies students in need of support through early needs identification and whole class identification. The OSS offers individualised learning skills sessions, peer-tutoring for high-risk subjects, interventions for students from low-resourced school backgrounds, and mental health and wellness.

Given the need to improve the retention and throughput of South African medical students, it is imperative to understand the predictive validity of the NBT and NSC results as selection tools for entry into medical programmes. While many studies have investigated the predictive validity of the NSC [8, 26, 27] and the NBT [7, 9], and the combined predictive validity of the NBT and the NSC [15, 28] for student performance, such research has not been conducted for first-year South African medical students at Wits University. Understanding the link between students’ performance in the NBT and the first year of the MBBCh degree will provide a measure of whether the combination of the NSC and NBT can discriminate between students with the potential to succeed academically and those without, to identify students who will require a foundation programme, and to identify academically at-risk students who may need additional support in the regular MBBCh programme. Focusing on first-year medical students enrolled at Wits University between 2011 and 2017, this study investigated the proportion of the variance in the academic success (passing the first year at the first attempt) explained by the three NBT domains and the four NSC subjects used in the admissions process. The study also sought to identify which of the NBT domains and NSC subjects used for selecting students are significant predictors for academic success. The study further explored the association between the NBT performance levels and students’ first-year progression outcome (passed, failed, or cancelled). Lastly, the link between students’ academic performance and school quintiles was investigated. The school quintiles were used as a proxy for socio-economic status, to understand how the disparities existing in the South African education systems influence students’ academic performance.

## Methods

This retrospective study analysed data for 1652 students registered for the MBBCh degree between 2011 and 2017, obtained from the Business Intelligence Services unit at Wits University. The data were analysed using IBM SPSS V25.0.

Frequency tables were used to describe the cohort’s demographic details and NBT performance. Crosstabs were used to understand the differences in first-year students’ progression outcomes (passed, failed, or cancelled) by school quintile.

A hierarchical regression was used to explore the amount of the variance in academic success explained by the three NBT domains while controlling for the four NSC subjects used for selection purposes. The independent variables were the results of three NBT domains and the four NSC subjects. The dependant variable was the first-year grade point average of students who passed. The grade point average is derived from the marks allocated for the first-year subjects in the MBBCh degree (see Table 4).

The hierarchical regression model included 1236 of the 1652 students in the study cohort who met the criteria for inclusion; namely, they had NBT results, NSC results for the required subjects, and had passed the first year. Of the 416 students who were removed from the regression analysis, 10 did not have NBT results, 157 did not have NSC results [Life Sciences (140), Physical Sciences (15) and English (2)], and 234 were students who had either discontinued the first year of study (89) or had failed the year (145). Before interpreting the results, the data were examined for the assumptions of the hierarchical regression (normality, linearity, intercorrelations, homoscedasticity and Mahalanobis distance) [29–31] and 15 outliers (students who fell above or below the interquartile range) were removed from the data used in the regression. ANOVA *F* statistics were used to confirm the predictive utility of the entire model. The *R*^2^ coefficients, which are the accurate proportion of the variation in the dependent variable, were interpreted and reported for the two models. The unstandardised (*B*) and standardised (β) regression coefficient and significance levels for the unique contribution of each predicting variable were examined to identify which variables were significant predictors of academic success in the first year of study. Lastly, the effect size of the model was calculated [32].

Chi-square tests were used to explore the association between students’ NBT performance levels and their first-year progression outcome (passed, failed, or cancelled). Of the 1652 students in the study cohort, ten did not have NBT results and were excluded from the analysis, leaving 1642 cases. Where cells in the contingency table had less than 5% frequency counts, the NBT levels were aggregated to increase the expected counts for the cells. Cell counts of less than 5% violate the assumption of chi-square tests necessary to avoid erroneously interpreting a result as significant (type I error) and incorrectly interpreting a result as not significant (type II error) [33]. The frequencies were raised above the critical 5% frequency by merging the following levels: NBT MAT *intermediate lower* and *basic*; NBT AL *intermediate upper* and *intermediate lower*; and NBT QL *intermediate lower* and *basic*. The Pearson chi-square value and the *p-*value ≤0.05 were used to assess the statistical significance of the association between each of the NBT domains and the first-year progression outcome. The effect size of the association was calculated and reported using Cohen’s conventions [32].

Ethics approval for the study was obtained from the Human Research Ethics Committee (Medical) of Wits University (Clearance Certificate Number M170490).

## Results

Table 5 shows that the students in the study cohort were predominantly female (56%; *n* = 931), Black^4^ (44%; *n* = 732), and of urban origin (78%; *n* = 1295). More than 80% had attended the better-resourced schools: 74% (1223) were from quintile 5 schools, and 7% (115) had attended quintile 4 schools.

**Table 5:** Student demographics (*N* = 1652)

Variables	2011	2012	2013	2014	2015	2016	2017
Race^4^							
Black	71 (38.8%)	136 (48.6%)	104 (47.1%)	99 (40.6%)	97 (40.2%)	110 (49.3%)	115 (44.2%)
White	53 (29.0%)	85 (30.4%)	65 (29.4%)	61 (25.0%)	68 (28.2%)	46 (20.6%)	64 (24.6%)
Coloured	14 (7.7%)	11 (3.9%)	8 (3.6.0%)	17 (7.0%)	18 (7.5%)	12 (5.4%)	21 (8.1%)
Indian	42 (23.0%)	45 (16.1%)	43 (19.5%)	65 (26.6%)	56 (23.2%)	55 (24.7%)	60 (23.1%)
Chinese	3 (1.6%)	3 (1.1%)	1 (0.5%)	2 (0.8%)	2 (0.8%)	0 (0.0%)	0 (0.0%)
Gender							
Male	67 (37.0%)	101 (36.0%)	94 (42.5%)	103 (42.0%)	113 (47.0%)	113 (51.0%)	130 (50.0%)
Female	116 (63.0%)	179 (64.0%)	127 (57.5%)	141 (58.0%)	128 (53.0%)	110 (49.0%)	130 (50.0%)
Origin							
Rural	14 (8.0%)	22 (8.0%)	23 (10.4%)	27 (11.1%)	66 (27.0%)*	67 (30.0%)*	62 (24.0%)*
Urban	149 (81.0%)	237 (85.0%)	191 (86.4%)	211 (86.5%)	164 (68.0%)	151 (68.0%)	192 (74.0%)
Unknown	20 (11.0%)	21 (7.0%)	7 (3.2%)	6 (2.5%)	11 (5.0%)	5 (2.0%)	6 (2.0%)
School quintile							
1	0 (0.0%)	3 (1.0%)	4 (2.0%)	1 (0.4%)	7 (3.0%)	18 (8.0%)	14 (5.0%)
2	6 (3.3%)	2 (5.0%)	11 (1.0%)	10 (4.1%)	17 (7.0%)	13 (6.0%)	28 (11.0%)
3	5 (2.7%)	10 (4.0%)	18 (8.0%)	8 (3.3%)	23 (9.5%)	25 (11.0%)	15 (6.0%)
4	9 (5.0%)	20 (7.0%)	16 (7.0%)	23 (9.4%)	18 (7.0%)	13 (6.0%)	16 (6.0%)
5	143 (78.1%)	224 (80.0%)	165 (75.0%)	196 (80.3%)	165 (68.5%)	149 (67.0%)	181 (70.0%)
Unknown	20 (11.0%)	21 (7.5%)	7 (3.0%)	6 (2.5%)	11 (5.0%)	5 (2.0%)	6 (2.0%)

* Wits University introduced rurality as a selection criterion in 2015

### First-year progression outcome for school quintile

**Fig. 1:**
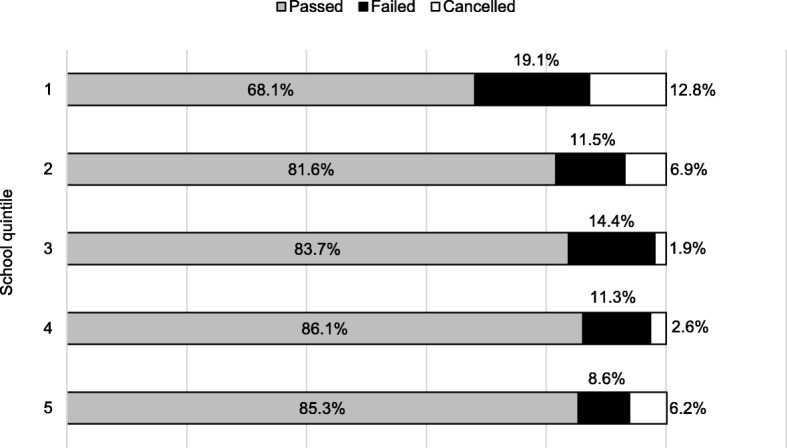
First-year progression outcome for school quintile

Figure 1 shows the first-year performance for the school quintiles. The pass rates for all quintiles were above 60%. Schools in quintile 1 were the worst-performing, with the lowest pass rate of 68.1% (32), the highest failure rate of 19.1% (9) and a cancellation rate of 12.8% (6). More than 80% of the students from schools in quintiles 2 to 5 passed the first year. Quintile 5 schools had the lowest failure rate of 8.6% (105), with a higher cancellation rate, 6.2% (75), than quintile 3 schools, 1.9% (2), and quintile 4 schools, 2.6% (3). The higher cancellation rate for students from quintile 5 schools could be explained by this quintile constituting the majority (74%, 1223) of the 1652 students in the study cohort.

Table 6 shows the NBT results for the study cohort. Only 54.3% (891) of the students with NBT MAT results were proficient. The NBT MAT domain had the highest number of students who fell outside of the *proficient* level 45.7% (751), followed by the NBT QL domain, 36.7% (606), and the NBT AL domain 23.8% (380).

**Table 6:** MBBCh students’ performance level for each NBT domain (2011–2017)

Performance levels	NBT MAT (*n* = 1642)*	NBT AL (*n* = 1646)*	NBT QL (*n* = 1646)*
Proficient	891 [54.3%]	1253 [76.1%]	1037 [63.0%]
Intermediate Upper	503 [30.6%]	313 [18.9%]	467 [28.4%]
Intermediate Lower	233 [14.2%]	80 [4.8%]	138 [8.4%]
Basic	15 [0.9%]		4 [0.2%]

*Number of students with results

### Predictive validity of the NBT and NSC

Table 7 shows the mean and standard deviation for the variables used in the regression. The correlation table in Additional file 1 shows a positive correlation amongst the independent variables of less than 0.7 and that no independent variable correlated with the dependant variable with a correlation coefficient of less than 0.3, as required for regression analyses.

**Table 7:** Descriptive Statistics (*N* = 1236)

	Mean	Std. Deviation
**First-year results**	65.59	7.773
**NBT Mathematics**	71.38	14.607
**NBT Academic Literacy**	73.79	9.476
**NBT Quantitative Literacy**	73.81	12.258
**NSC English**	80.98	6.390
**NSC Maths**	87.18	7.566
**NSC Life Sciences**	85.50	5.355
**NSC Physical Science**	83.87	8.356

The NBT domains explained a statistically significant 26% of variance of the first-year grade point average of the students who passed, *R*^*2*^ = 0.263, *F* (3, 1232) = 146.78, *p* < 0.000. After controlling for the role of the NBT domains, the four NSC subjects, Life Sciences, English, Mathematics, and Physical Science, explained 19% of the variance, *R*^*2*^ = 0.188, *F* (3, 1229) = 137.14, *p* < 0.000. In combination, all predicting variables contributed 45% of the variance in the final marks for the students who passed the first year of study at the first attempt, *R*^*2*^ = 0.451, *F* (6, 1229) = 166.29, *p* < 0.000. The unstandardised (*B*) and standardised (β) regression coefficient and squared semi-partial correlations (*sr*^*2*^) (which denotes the significance of each variable) for the unique contribution of each predicting variable are reported in Table 8. The effect size of this model was (*f*^2^ = 0.82).

**Table 8:** Significance of each predictor to first-year academic success

Variable	B [95% CI]	***β***	Sig
**Model 1**			
**NBT Mathematics**	0.177 [0.146, 0.207]	0.332	0.000**
**NBT Academic Literacy**	0.142 [0.095, 0.190]	0.174	0.000**
**NBT Quantitative Literacy**	0.078 [0.037, 0.120]	0.124	0.000**
**Model 2**			
**NBT Mathematics**	0.009 [−0.024, 0.041]	0.016	0.596
**NBT Academic Literacy**	0.171 [0.125, 0.216]	0.208	0.000**
**NBT Quantitative Literacy**	0.068 [0.032, 0.104]	0.107	0.000**
**English**	0.081 [0.019, 0.144]	0.067	0.011*
**Maths**	0.263 [0.193, 0.333]	0.256	0.000**
**Life Sciences**	0.199 [0.118, 0.280]	0.137	0.000**
**Physical Science**	0.177 [0.116, 0.238]	0.190	0.000**

*CI* confidence interval.

*p < 0.01, **p < 0.000

The lack of significance of the NBT MAT domain could be attributed to the overlap between the content assessed in this domain and the mathematics content taught in Grade 12, as required by the Curriculum and Assessment Policy Statement for Mathematics [34].

### Association between the NBT levels and student performance

**Fig. 2:**
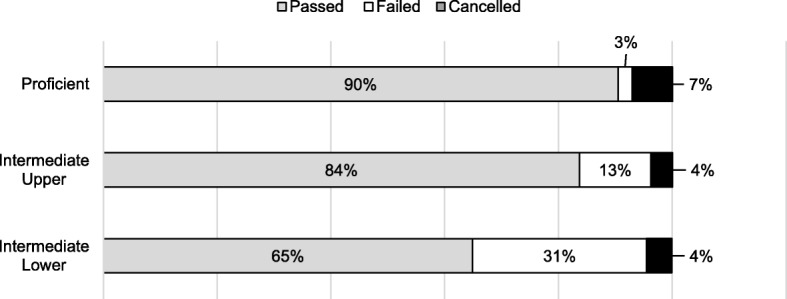
Associations between NBT MAT levels and first-year progression outcomes

A Pearson’s chi-square test between the NBT MAT proficiency levels and the first-year progression outcome was statistically significant, χ^2^ (4, *N* = 1642) = 184.513, *p* = 0.000. The association between the NBT MAT and first-year progression outcome was small, φ = 0.23. Figure 2 shows that students who were proficient in the NBT MAT were more likely to pass the first year compared to students admitted with results at the *intermediate upper* and *intermediate lower* levels.

**Fig. 3:**
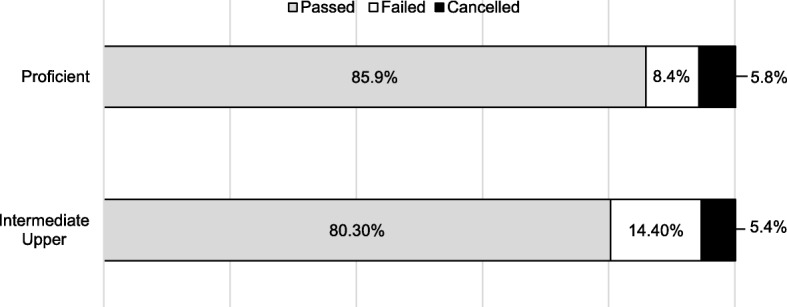
Associations between NBT AL performance levels and first-year progression outcomes

The *intermediate upper* and *intermediate lower* levels were aggregated to increase the counts for the NBT AL levels. A Pearson’s chi-square test between the NBT AL proficiency levels and the first-year overall outcome was statistically significant, χ^2^ (2, *N* = 1642) = 11.994, *p* = 0.002. The association between the NBT AL and first-year progression outcome was small, φ = 0.08. As shown in Fig. 3, students who were proficient in the NBT AL domain performed better than students who obtained results in both intermediate levels.

**Fig. 4:**
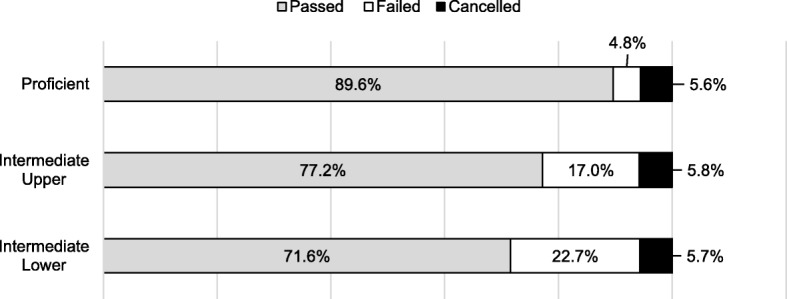
Associations between NBT QL and first-year progression outcomes

In the NBT QL domain, the *intermediate lower* and *basic* levels were combined to provide frequencies greater than 5%. A Pearson’s chi-square test between the NBT QL proficiency levels and first-year results was statistically significant, χ^2^ (4, *N* = 1642) = 83.433, *p* = 0.002. The association between the NBT QL and first-year progression outcome was small, φ = 0.159. Despite the small effect size, students who were proficient performed academically better compared to students in either the intermediate upper or the intermediate lower level, as shown in Fig. 4.

## Discussion

Of the seven variables tested in the regression, six were statistically significant in predicting academic success in the first year of study, the four NSC subjects and the two of the three NBT domains, the NBT QL and NBT AL. The NBT MAT domain was not statistically significant in this study, raising the question of whether Wits University and other universities using the NBT for selection purposes, need to reconsider the weighting of the NBT MAT domain. The NBT accounted for a greater amount of the variance in predicting academic success in the first year of study than the NSC. This finding, together with the strong association between the NBT proficiency levels and the first-year progression outcome that was observed, suggests that the NBT should be used together with the NSC to strengthen the admissions process. The student performance by school quintile showed that students who attended lower quintile schools were more likely to experience academic difficulties than those who attended schools in more affluent communities. This raises questions about whether adequate support was provided to the large number of students admitted with low entry-level skills.

Other authors have advocated that an additional selection test is used to complement the NSC. For example, Wadee and Cliff [4] advocated the use of more than one selection tool after they found that the NSC subjects correlate weakly with the first-year academic performance of medical students at Wits University. The NSC subjects were also found to be poor predictors of academic success in the first year of study in a Bachelor of Optometry programme [26]. After tracking the results of a cohort of students from three faculties over 6 years (2009–2014) at another South African higher-education institution, Prince [11] called for the combined use of the NBT with the NSC to improve student placement and retention. Our findings provide empirical evidence supporting the combined use of the NBT and NSC results for the selection of medical students. In our study, the NSC only accounted for 19% of the variance in predicting academic success in the first year of the medical programme. The greater variance (26%) explained by the NBT supports its use as an additional tool to complement the NSC as a predictor of academic success in the first year of study. However, only five of the nine medical schools in South Africa use a combination of the two tools, with other medical schools weighting the NBT lower than 50%.

In addition to the capacity of the NBT to assess students’ abilities to engage with tertiary-level education, the NBT allows the identification of students who may need to be placed into suitable foundation programmes, based on academic skills in which they lack proficiency, and for identifying those admitted to the regular MBBCh programme who may need additional support. The norm-referenced basis of the NSC results, by contrast, offer limited potential for identifying students in medical programmes who need academic support [4, 11]. We observed a strong association between the proficiency levels of the three NBT domains and students’ first-year progression outcomes. Despite the low effect sizes for the associations, students who were proficient in the NBT domains were more likely to perform well academically, raising the question of what support is available for students who are admitted with low entry-level skills. The recent adjustments in university admission policies to include more students from lower quintile schools and rural areas [3] have increased the proportion of students with low entry-level skills [9]. The disparities between students’ entry-level skills and university admission requirements could be attributed to the stark differences between lower-quintile and higher-quintile schools [20–22], which reflect the socio-economic differences in the country. While it is in the interests of social justice to admit students who reflect the demographics of the country [3], our results suggest that there is a higher probability that students with low entry-level skills need adequate support to cope with the academic demands of medical education.

Foundation programmes play a crucial role in addressing the learning disparities existing in secondary education and also in promoting students’ chances of continued academic success [35]. Some authors [11, 20, 36] have advocated for the use of foundation programmes to address historical disadvantages in education and socio-economic background in countries like Australia, the United Kingdom, and South Africa. Students placed in foundation programmes are more likely to succeed in the first year of study [11, 36, 37]. Most South African higher-education students exceed the minimum time allowed to complete their studies [11, 19]. Foundation programmes could improve the first-year success rate, which, ultimately, will improve retention and throughput rates. The academic demands of the medical programme may be a huge adjustment for students with low entry-level capabilities [11]. Based on their NBT results, a large proportion of students in this study who failed the first year at their first attempt could have benefitted from additional support, possibly in the form of a foundation programme. Nearly 31% of the students from quintile 1 (19.1%; 9) and quintile 2 schools (11.5%; 10) failed, and close to 20% from quintile 1 (12.8%; 6) and quintile 2 (6.9%; 6) cancelled their studies. The NBT recommends additional support for students accepted with results at the *upper intermediate* level, placement in foundation programmes for students with results at the *intermediate lower* level, and placement in other programmes for students with results at the *basic* level. More than 45.7% of the study cohort were accepted with NBT results that fell outside of the *proficient* level for the NBT MAT domain alone, while 0.9% of the students were accepted with NBT MAT results at the *basic* level. As access to medical education in South Africa is widened to accommodate students from different backgrounds and with different entry-level skills, foundation programmes could be useful to improve retention and throughput [36]. In 2016, van der Merwe et al. [3] reported that five of the nine South African medical schools offered foundation programmes, but perhaps other medical schools need to consider this.

The NBT and NSC investigated in this study only accounted for 45% of the variance in the academic success of the first-year students. This leaves 55% of the variance that could be attributed to other factors that have an impact on students’ academic performance. For example, Ahmady et al. [38] found that students’ learning styles, level of motivation, and being first-generation students influenced their performance. Arulampalam [39] reported on the influence of the location of students’ accommodation on their academic achievement, while Yorke [40] explored the impact of their career choice, financial difficulties encountered at home and while at university, and the strategies used in medical teaching. Another factor influencing student academic success is the types of support available. We have reported on the types of support available to medical students at Wits University, but we have not reported on what support students made use of and to what extent.

The myriad factors impacting on student performance in medical degrees, especially the widening of access to include more students from remote areas and students from disadvantaged backgrounds, provide opportunities for further study, both globally and locally, to promote equitable access that fosters fair chances for success. At a local level, areas for future research include investigating the relationship between students’ scores for different NBT domains and their academic performance, the impact of different types of support on student achievement, and the impact of foundation programmes on student retention and throughput.

## Conclusion

These results suggest that the NBT, when weighted equally with the NSC results, explains more variance than the NSC results alone in predicting students’ academic success in the first year of medical study. Based on the stronger predictive validity of the NBT, South African medical schools should implement the NBT as an additional selection tool to the NSC, with an equivalent weighting. We further recommend that the NBT, which was commissioned to assess students’ readiness for university, should not be used only as a selection tool for admissions, but also to identify students likely to require additional support, possibly through foundation programmes. Our findings may be applicable to other South African medical schools and other health science professional programmes.

## Supplementary information

**Additional file 1.**

## Data Availability

The datasets used and analysed during the current study are available from the corresponding author on reasonable request.

## Abbreviations

NSC: National Senior Certificate
NBT: National Benchmark test
NBT MAT: National Benchmark Test in Mathematics
NBT AL: National Benchmark in Test Academic Literacy
NBT QL: National Benchmark Test in Quantitative Literacy
Wits University: University of the Witwatersrand
OSS: Office of Student Success
MBBCh: Bachelor of Medicine and Bachelor of Surgery

## Footnotes

1 The South African Education system is divided into the Basic Education and Higher Education systems. The Basic Education system consists of the General Education Training level (grades R-9) and the Further Education Training level (grades 10–12). Students write the NSC examination at the end of the grade 12 year of the Further Education Training level.

2 Higher Education South Africa, an organisation representing South Africa’s universities, became ‘Universities South Africa’ in 2015.

3 The term ‘ordinary public’ distinguishes government schools classified according to the quintile system from Schools of Specialisation that offer specialised curricula and are excluded from the quintile system.

4 The racial groupings of Black, Coloured (mixed race), and Indian, were introduced in South Africa during the apartheid era (1947–1994) according to the Population Registration Act (No. 30 of 1950). The terms are still in use to address the racial inequities in the country. Chinese people were classified as either Coloured or Indian. Where relevant, Chinese people are reported as a separate group in this paper.
