# Supplementary material for: Predictive validity of the National Benchmark Test and National Senior Certificate for the academic success of first-year medical students at one South African university
Source: BMC Med Educ. 2020 May 13;20:152. doi: 10.1186/s12909-020-02059-8 (PMC7218523; doi:10.1186/s12909-020-02059-8)
Supplement: Supplementary file 1 — Additional file 1. [file 12909_2020_2059_MOESM1_ESM.docx]

| **Correlations** | | | | | | | | | |
| --- | --- | --- | --- | --- | --- | --- | --- | --- | --- |
|  | | NBT Mathematics | NBT Academic Literacy | NBT Quan-titative Literacy | English | Maths | Life Science | Physical Science | First-year results |
| NBT Mathematics | Pearson Correlation | 1 | .341^**^ | .542^**^ | .268^**^ | .643^**^ | .337^**^ | .441^**^ | .458^**^ |
|  | Sig. (2-tailed) |  | .000 | .000 | .000 | .000 | .000 | .000 | .000 |
|  | N | 1236 | 1236 | 1236 | 1236 | 1236 | 1236 | 1236 | 1236 |
| NBT Academic  Literacy | Pearson Correlation | .341^**^ | 1 | .562^**^ | .426^**^ | .150^**^ | .109^**^ | .002 | .356^**^ |
|  | Sig. (2-tailed) | .000 |  | .000 | .000 | .000 | .000 | .941 | .000 |
|  | N | 1236 | 1236 | 1236 | 1236 | 1236 | 1236 | 1236 | 1236 |
| NBT Quantitative Literacy | Pearson Correlation | .542^**^ | .562^**^ | 1 | .273^**^ | .368^**^ | .135^**^ | .194^**^ | .401^**^ |
|  | Sig. (2-tailed) | .000 | .000 |  | .000 | .000 | .000 | .000 | .000 |
|  | N | 1236 | 1236 | 1236 | 1236 | 1236 | 1236 | 1236 | 1236 |
| English | Pearson Correlation | .268^**^ | .426^**^ | .273^**^ | 1 | .336^**^ | .426^**^ | .306^**^ | .391^**^ |
|  | Sig. (2-tailed) | .000 | .000 | .000 |  | .000 | .000 | .000 | .000 |
|  | N | 1236 | 1236 | 1236 | 1236 | 1236 | 1236 | 1236 | 1236 |
| Maths | Pearson Correlation | .643^**^ | .150^**^ | .368^**^ | .336^**^ | 1 | .491^**^ | .694^**^ | .559^**^ |
|  | Sig. (2-tailed) | .000 | .000 | .000 | .000 |  | .000 | .000 | .000 |
|  | N | 1236 | 1236 | 1236 | 1236 | 1236 | 1236 | 1236 | 1236 |
| Life Sciences | Pearson Correlation | .337^**^ | .109^**^ | .135^**^ | .426^**^ | .491^**^ | 1 | .619^**^ | .452^**^ |
|  | Sig. (2-tailed) | .000 | .000 | .000 | .000 | .000 |  | .000 | .000 |
|  | N | 1236 | 1236 | 1236 | 1236 | 1236 | 1236 | 1236 | 1236 |
| Physical Sciences | Pearson Correlation | .441^**^ | .002 | .194^**^ | .306^**^ | .694^**^ | .619^**^ | 1 | .501^**^ |
|  | Sig. (2-tailed) | .000 | .941 | .000 | .000 | .000 | .000 |  | .000 |
|  | N | 1236 | 1236 | 1236 | 1236 | 1236 | 1236 | 1236 | 1236 |
| YOS1 | Pearson Correlation | .458^**^ | .356^**^ | .401^**^ | .391^**^ | .559^**^ | .452^**^ | .501^**^ | 1 |
|  | Sig. (2-tailed) | .000 | .000 | .000 | .000 | .000 | .000 | .000 |  |
|  | N | 1236 | 1236 | 1236 | 1236 | 1236 | 1236 | 1236 | 1236 |
| ** Correlation is significant at the 0.01 level (2-tailed) | | | | | | | | | |

| **Correlations** | | | | | | | | |
| --- | --- | --- | --- | --- | --- | --- | --- | --- |
|  | | First-year results | NBT AL | NBT QL | NBT MAT | Maths | Life Science | Physical Science |
| Pearson Correlation | First-year results | 1.000 | .356 | .401 | .458 | .559 | .452 | .501 |
|  | NBT AL | .356 | 1.000 | .562 | .341 | .150 | .109 | .002 |
|  | NBT QL | .401 | .562 | 1.000 | .542 | .368 | .135 | .194 |
|  | NBT MAT | .458 | .341 | .542 | 1.000 | .643 | .337 | .441 |
|  | Maths | .559 | .150 | .368 | .643 | 1.000 | .491 | .694 |
|  | Life Science | .452 | .109 | .135 | .337 | .491 | 1.000 | .619 |
|  | Physical Science | .501 | .002 | .194 | .441 | .694 | .619 | 1.000 |
| Sig. (1-tailed) | First year result | . | .000 | .000 | .000 | .000 | .000 | .000 |
|  | NBTAL | .000 | . | .000 | .000 | .000 | .000 | .470 |
|  | NBTQL | .000 | .000 | . | .000 | .000 | .000 | .000 |
|  | NBT MAT | .000 | .000 | .000 | . | .000 | .000 | .000 |
|  | Maths | .000 | .000 | .000 | .000 | . | .000 | .000 |
|  | Life Science | .000 | .000 | .000 | .000 | .000 | . | .000 |
|  | Physical Science | .000 | .470 | .000 | .000 | .000 | .000 | . |
| N | First-year results | 1236 | 1236 | 1236 | 1236 | 1236 | 1236 | 1236 |
|  | NBTAL | 1236 | 1236 | 1236 | 1236 | 1236 | 1236 | 1236 |
|  | NBTQL | 1236 | 1236 | 1236 | 1236 | 1236 | 1236 | 1236 |
|  | NBT MAT | 1236 | 1236 | 1236 | 1236 | 1236 | 1236 | 1236 |
|  | Maths | 1236 | 1236 | 1236 | 1236 | 1236 | 1236 | 1236 |
|  | Life Science | 1236 | 1236 | 1236 | 1236 | 1236 | 1236 | 1236 |
|  | Physical Science | 1236 | 1236 | 1236 | 1236 | 1236 | 1236 | 1236 |
